# The Roles of Histone Deacetylases in the Regulation of Ovarian Cancer Metastasis

**DOI:** 10.3390/ijms242015066

**Published:** 2023-10-11

**Authors:** Long Xu, Xiaoyu Yan, Jian Wang, Yuanxin Zhao, Qingqing Liu, Jiaying Fu, Xinyi Shi, Jing Su

**Affiliations:** 1 Key Laboratory of Pathobiology, Department of Pathophysiology, Ministry of Education, College of Basic Medical Sciences, Jilin University, 126 Xinmin Street, Changchun 130021, China; xulong20@mails.jlu.edu.cn (L.X.); yanxy@jlu.edu.cn (X.Y.); wjian21@mails.jlu.edu.cn (J.W.); zhaoyx19@mails.jlu.edu.cn (Y.Z.); liuqq22@mails.jlu.edu.cn (Q.L.); fujy21@mails.jlu.edu.cn (J.F.); shixy9921@mails.jlu.edu.cn (X.S.); 2 School of Medicine, Southern University of Science and Technology, Shenzhen 518000, China

**Keywords:** ovarian cancer, metastasis, histone deacetylases, histone deacetylase inhibitors

## Abstract

Ovarian cancer is the most lethal gynecologic malignancy, and metastasis is the major cause of death in patients with ovarian cancer, which is regulated by the coordinated interplay of genetic and epigenetic mechanisms. Histone deacetylases (HDACs) are enzymes that can catalyze the deacetylation of histone and some non-histone proteins and that are involved in the regulation of a variety of biological processes via the regulation of gene transcription and the functions of non-histone proteins such as transcription factors and enzymes. Aberrant expressions of HDACs are common in ovarian cancer. Many studies have found that HDACs are involved in regulating a variety of events associated with ovarian cancer metastasis, including cell migration, invasion, and the epithelial–mesenchymal transformation. Herein, we provide a brief overview of ovarian cancer metastasis and the dysregulated expression of HDACs in ovarian cancer. In addition, we discuss the roles of HDACs in the regulation of ovarian cancer metastasis. Finally, we discuss the development of compounds that target HDACs and highlight their importance in the future of ovarian cancer therapy.

## 1. Introduction

Ovarian cancer is the second most common cause of death in women with gynecologic cancers [1]. It is estimated that there will be approximately 19,710 new ovarian cancer cases, and 13,270 deaths occurred in the United States in 2023 [2]. Epithelial ovarian cancer is the most common pathological type of ovarian cancer, accounting for more than 90% of all malignant ovarian tumors [3]. Due to the lack of effective screening strategies and a lack of specific symptoms at the early stage of ovarian cancer, most patients are diagnosed at an advanced stage when the tumor has metastasized throughout the peritoneal cavity [4]. Tumor metastasis is closely associated with the poor prognosis of ovarian cancer patients and is also the main cause of death in patients with ovarian cancer [5].

Epigenetic processes may play important roles in the development and progression of ovarian cancer [6]. Histone acetylation is one of the most well-studied epigenetic processes. Besides histones, many non-histone proteins can also be acetylated [7]. Histone acetylation plays an important role in the regulation of gene transcription. In general, increased histone acetylation is associated with chromatin relaxation, which can facilitate gene transcription. In contrast, decreased histone acetylation inhibits gene expression [8]. In addition to histones, acetylation often occurs on a variety of non-histone proteins [9]. The balance between acetylation and deacetylation is regulated by histone acetyltransferases (HATs) and histone deacetylases (HDACs). Aberrant expressions of HDACs have been found in a variety of tumors, including those of ovarian cancer [10,11,12]. A dysregulated expression of HDACs in ovarian cancer would lead to an imbalance between the acetylation and deacetylation of histone or non-histone proteins and participate in the development and progression of ovarian cancer through regulating a variety of key cellular processes, including immune response, DNA repair, cell cycle, metastasis and angiogenesis [13].

Given their important roles in gene expression, cell proliferation, apoptosis, metabolism and tumor metastasis, HDACs have become a promising drug target for tumor therapy. Since the first pan-HDAC inhibitor vorinostat (SAHA) was approved by the United States Food and Drug Administration (FDA), HDACs, including sirtuins (SIRT1–7), have received great attention [14,15]. A large number of studies have found that HDAC inhibitors (HDACIs) can significantly inhibit cancer cell proliferation and metastasis, which may be related to differentiation, immune regulation, chromatin instability, DNA damage repair, oxidative stress, cell cycle arrest, autophagy and angiogenesis [16]. In this review, we focus mainly on the roles of HDACs in ovarian cancer metastasis and the research progress of HDACIs in ovarian cancer therapy.

## 2. Ovarian Cancer

Ovarian cancer is the most lethal gynecologic malignancy and consists of a series of tumors with different developmental origins, histopathological features, genetic alterations, clinical behaviors and molecular profiles [17,18,19]. According to the dualistic model of carcinogenesis, ovarian cancer can be classified as type I or type II [20]. Type I tumors include clear-cell, endometrioid, mucinous, and low-grade serous ovarian cancer. These tumors are chromosomally stable, show few p53 mutations, and have a favorable prognosis [21]. Type II tumors comprise high-grade serous ovarian cancer, undifferentiated carcinomas and carcinosarcomas. Type II tumors are more aggressive, constituting 75% of ovarian cancers with a 90% death rate. In addition, these tumors generally have gross chromosomal instability and commonly possess mutations in p53 [22,23]. In addition to gene mutations, significant histotypespecific epigenetic changes are characterized between the different histotypes and when compared to normal tissues [24]. For example, global decreased DNA methylation (hypomethylation) is common across all histotypes of ovarian cancer. High-grade serous ovarian cancer is distinct from low-grade serous ovarian cancer based on methylation patterns, and is more hypomethylated than clear-cell ovarian cancer and endometrioid ovarian cancer [25]. In addition, chromatin alterations between different histotypes have also been beginning to be characterized [24]. Currently, the standard treatment for ovarian cancer is maximal cytoreductive surgical debulking followed by platinum-based chemotherapy [26]. In the early stage of ovarian cancer, when the tumor is confined to one or both ovaries, ovarian cancer is curable and less than 10% of patients will die of ovarian cancer [27]. Unfortunately, the majority of patients present a stage III/IV tumor and the five-year survival rate of patients is less than 30% [1]. Advanced ovarian cancer is characterized by peritoneal cavity and/or retroperitoneal lymph node metastasis and an extensive spread of the tumor beyond the abdomen [28]. In addition, approximately 90% of patients with advanced ovarian cancer also develop peritoneal cancer and malignant ascites [29]. Debulking surgery and chemotherapy are less effective when the tumor has metastasized to distant organs [4]. Tumor metastasis is the major cause of cancer morbidity and mortality, accounting for approximately 90% of cancer-related deaths [30]. Therefore, further in-depth studies on the metastatic potential of primary ovarian cancer are needed to promote the development of ovarian cancer therapies and the optimization of the management of ovarian cancer patients.

Tumor metastasis is a multistep process. In this process, tumor cells spontaneously or passively detach, shed and spread from the primary tumor to surrounding tissues and distant organs [31]. Ovarian cancer metastasis can proceed through several different mechanisms, including transcoelomic, hematogenous and lymphogenous [27]. Unlike other well-documented cancers that spread primarily through the hematogenous route, transcoelomic metastasis is the most important route in ovarian cancer [32]. The first step of transcoelomic metastasis is to disseminate from the primary ovarian cancer tumor through epithelial–mesenchymal transformation (EMT) [33]. Once cancer cells depart from the primary site, they float freely as spheroids in the peritoneal ascites. The metastatic cells then attach to the mesothelial lining or invade deeper into the peritoneal organs [27]. During this process, adipocytes promote ovarian cancer metastasis and support tumor growth [34,35]. In addition, metastatic ovarian cancer cells can invade through the blood or lymphatic vessels to distant sites and establish new tumors in hematogenous and lymphatic metastases [27,36]. It is widely accepted that hematogenous metastasis is of limited importance in ovarian cancer metastasis. However, emerging evidence indicates that high-grade serous ovarian cancer arising from the fallopian tube preferentially spreads to the ovary via hematogenous metastasis [37]. In addition, circulating tumor cells, which are tumor cells that have sloughed off the primary tumor and extravasate into and circulate in the blood, have long been considered as an effective indicator of hematogenous metastasis in a variety of solid tumors as well as in ovarian cancer [38,39,40]. Therefore, we should pay more attention to the roles of the hematogenous route in ovarian cancer metastasis.

Imbalances in histone post-translational modifications, particularly histone acetylation, are common in human cancers, which would lead to imbalances in gene transcription [41]. Aberrant histone H4 Lys16 acetylation is a common event in human cancers as well as ovarian cancer [42,43]. In human tumor tissues, decreased levels of histone acetylation significantly correlated with tumor malignancy and tumor invasion [44,45]. The level of histone acetylation in the deeply invasive part of the tumor was significantly lower than that in the superficial part of tumor, suggesting that global histone deacetylation may be involved in tumor cell invasion and metastasis [46]. As histone and non-histone acetylation play an important role in tumorigenesis and metastasis, further in-depth studies on enzymes regulating protein acetylation may be beneficial to the development of ovarian cancer therapies.

## 3. Histone Deacetylases

There are extensive epigenetic changes in ovarian cancer, which are closely related to the development, metastasis and heterogeneity of ovarian cancer [8]. The epigenetic modifications include chromatin remodeling, histone modification, DNA methylation, and noncoding RNA expression [47]. The aberrant regulation of histone modifications, such as methylation, acetylation, and monoubiquitination, are common in ovarian cancer [48]. Histone acetylation is one of the best studied histone modification mechanisms, which is carefully controlled by HATs and HDACs [47,49]. In general, HATs relax the chromatin by neutralizing the positive charge of histone lysine residues, which facilitates gene transcription. In contrast, HDACs deacetylate certain histones, which inhibits gene transcription [50]. To date, 18 kinds of HDACs have been found in mammalian cells. Based on sequence similarity to yeast deacetylases, HDACs can be divided into four major groups [51,52,53]. Class I HDACs include HDAC1, 2, 3, and 8, which are homologous to the yeast reduced potassium dependency 3 (Rpd3) protein. Class II HDACs are homologous to the yeast histone deacetylase 1 (Hda1) protein. Class II histone deacetylases can be further divided into class IIa (HDAC4, 5, 7, and 9) and class IIb (HDAC6 and HDAC10). SIRTs are class III histone deacetylases, which are homologous to yeast silent information regulator 2 (Sir2) and include seven isoforms (SIRT 1-7) [54,55]. HDAC11 is the only member of class IV HDAC. Class I, II and IV HDACs, also known as classical HDACs, are Zn^2+^-dependent. Although classical HDACs can be found in the cytoplasm and nucleus, their mitochondrial localization has not been found [7,56]. Class I and IV HDACs are located in the nucleus, class IIb histone deacetylases are located in the cytoplasm, and class IIa histone deacetylases are located in the nucleus in the basal state and can transport to the cytoplasm under the stimulation of certain signals [57,58]. SIRTs, which are NAD^+^-dependent, are located in different cell compartments, including the nucleus (SIRT1 and 6), nucleolus (SIRT7), cytoplasm (SIRT2) and mitochondria (SIRT3, SIRT4, and SIRT5) [59].

In addition to histones, HDACs can also regulate the acetylation of many non-histone proteins, such as transcription factors and enzymes [8]. The positively charged lysine residues frequently participate in protein–protein interactions and protein catalytic activity [9]. Acetylation can neutralize the positive charge carried by lysine residues and regulate protein function via a variety of mechanisms, including protein stability, enzymatic activity, subcellular localization, and interaction with other intracellular biomolecules [9,60]. Therefore, an aberrant expression of HDACs may lead to a disruption of the balance between the acetylation and deacetylation of histones and multiple non-histone proteins in cancer cells, affecting gene expression as well as the activity of numerous key proteins, which in turn affects tumor cell proliferation, apoptosis, cell cycle, invasion and migration [61,62].

## 4. Roles of Histone Deacetylases in Ovarian Cancer Metastasis

The overexpression of class I HDACs is common in metastatic high-grade carcinomas and tumors with distant metastases and plays a key role in accelerating metastasis [63]. In ovarian cancer, the expression of class I HDACs was positively correlated with the malignancy of the tumor, and a high expression of class I HDACs was an independent risk factor of the poor prognosis of patients with ovarian cancer [64,65]. In ovarian cancer cells, the expression of HDAC1 and HDAC2 was significantly positively correlated with the expression of Ki-67, which is essential for the proliferation of ovarian cancer cells. The expression of HDAC3 was negatively correlated with the expression of E-cadherin, which can affect the invasion and migration of ovarian cancer cells [66]. In addition, HDAC3 is able to promote cell proliferation, invasion and migration by activating the phosphoinositide 3-kinase (PI3K)/AKT signaling pathway [67]. In wild-type p53 ovarian cancer cells, HDAC8 inhibition can significantly inhibit cell invasion and migration, suggesting that HDAC8 may also be involved in the regulation of ovarian cancer metastasis [68].

HDAC6 are involved in the regulation of cell proliferation, cell motility, metastasis, mitosis and DNA repair, by deacetylating α-tubulin, cortactin, heat shock protein 90 (HSP90), P53, and MutS homologue-2 (MSH2) [69,70]. In wild-type p53 ovarian cancer cells, the inhibition of HDAC6 significantly represses cell proliferation, and suppresses cell migration [68]. HDAC6 is thought to play an important role in transformation induced by oncogene Ras, which is an important factor in tumorigenesis and the maintenance of the transformed phenotype and is involved in cancer invasion and metastasis. In addition, HDAC6 is required for the activation of the mitogen-activated protein kinase (MAPK) and the phosphoinositide 3 kinase (PI3K), which can facilitate anchorage-independent cell growth. HDAC6 may promote tumorigenesis by promoting the activation of Ras and its downstream PI3K and MAPK pathways [71]. HDAC9 has a histological subtype-specific effect on the prognosis of ovarian cancer patients. HDAC9 expression levels were negatively correlated with the prognosis of patients with high-grade serous ovarian cancer. In contrast, high expression levels of HDAC9 were associated with a higher survival rate for patients with non-serous ovarian cancer. In serous ovarian cancer cells, overexpressed HDAC9 can activate EMT and promote cell migration and invasion via increasing the nuclear localization of forkhead box O1 (FOXO1) and promoting the expression of the transforming growth factor β (TGFβ). In non-serous ovarian cancer cells, HDAC9 decreases the acetylation of β-catenin K49 and induces β-catenin translocation to the cytoplasm, inactivating EMT and inhibiting cell migration and invasion [72]. Intermediate filament family orphan 1 (IFFO1) can inhibit the nuclear accumulation of β-catenin, cancer metastasis and cisplatin resistance. An overexpression of HDAC5 in ovarian cancer cells inhibits the transcription of IFFO1 and enhances the proliferation, migration and chemoresistance of ovarian cancer cells [73].

SIRT1 can shuttle between the nucleus and the cytoplasm, and it seems that SIRT1 functions as a tumor suppressor or oncogene may depend on its subcellular localization. In ovarian cancer cells, cytoplasmic SIRT1 inhibits cell migration and invasion by impeding EMT. On the contrary, an overexpression of wild-type SIRT1 promotes cell migration and invasion [74]. In serous ovarian cancers, a reduced expression of SIRT2 can promote cell migration and invasion [75]. SIRT3 expression levels were significantly downregulated in the metastatic tissues and highly metastatic cell lines of ovarian cancer, and overexpressed SIRT3 inhibited EMT and ovarian cancer metastasis [76]. The expression of SIRT6 in ovarian cancer tissues was significantly higher than that in normal tissues. SIRT6 promotes the migration and invasion of ovarian cancer cells by inducing mitochondrial fission and promoting the formation of stress fibers [77]. SIRT7 is highly expressed in ovarian cancer tissues and cells, and silencing SIRT7 can inhibit the proliferation, invasion and migration of ovarian cancer cells [78].

HDAC11 is responsible for the deacetylation of core histones and is a key factor in the regulation of gene transcription and the cell cycle [79]. It has been reported that the loss of HDAC11 in ovarian cancer cells can inhibit the metabolic activity of cells and induce cell death [80]. However, the role of HDAC11 in ovarian cancer metastasis has been rarely reported.

## 5. The Underlying Mechanism of Histone Deacetylases in Regulating Ovarian Cancer Metastasis

EMT is a biological process in which ovarian cancer cells can diminish cell–cell adhesion and become more spindle-shaped mesenchymal cells with increased migratory capacities [81]. These more-mesenchymal cells generated via EMT can be reverted back to an epithelial state in the reverse process, known as the mesenchymal–epithelial transition (MET). Cancer cells can enter an intermediate partial EMT state with characteristics of both epithelial and mesenchymal cells as they rarely execute a complete EMT program that drives them into a fully mesenchymal state [81]. E-cadherin, encoded by the CDH1 gene, is the cornerstone of the epithelial state of the cell and the down-regulation of E-cadherin represents a hallmark of the activation of the EMT program. In ovarian cancer, a loss of E-cadherin is associated with tumor progression, an invasive characteristic of the tumor and the poor prognosis of patients [82,83]. Epigenetic regulators play an important role in the expression of E-cadherin and the epigenetic silencing of E-cadherin expression is a highly complex process. Multiple transcription factors and histone-modifying enzymes can negatively modulate E-cadherin transcripts by binding to the E-box elements of its promoter [84]. The nucleosome-remodeling and deacetylase (NuRD) complex plays important roles in the regulation of E-cadherin in multiple tumors including hepatocellular carcinoma and pancreatic cancer. EMT-inducing transcription factors (EMT-TFs) such as snail and twist can recruit the NuRD complex to the CDH1 promoter and inhibit E-cadherin expression by directly binding to the NuRD complex [85,86,87]. In ovarian cancer, the roles of the NuRD complex in the regulation of EMT have rarely been reported. The metastasis-associated (MTA) family members are the best studied subunits of the NuRD complex. Increased levels of MTA1 were subsequently observed in ovarian cancer, and the overexpression of MTA1 can promote cell invasion and metastasis [88,89]. Chromodomain helicase DNA binding protein 4 (CHD4) is another core component of the NuRD complex and mainly plays a role in cancer by participating in histone deacetylation and PARP-dependent DNA damage repair, which is frequently mutated in ovarian carcinomas [90]. CHD4 induces the suppression of the migration and invasion of ovarian cancer cells by suppressing the expression of EZH2 and the nuclear accumulation of β-catenin [91]. HDAC1/2 selective inhibitor romidepsin can suppress the progression of metastases in vitro and in vivo through inhibiting the functions of CHD4 [91]. This research suggest that the NuRD complex may also participate in regulating ovarian cancer metastasis, and further studies are needed to determine the mechanisms through which the NuRD complex regulates EMT and ovarian cancer metastasis.

EMT is also regulated by various EMT-TFs. The snail family is the best-studied EMT-TFs. In SGOCL cells, the expression of snail has a negative correlation with the expression of slug, where snail predominantly represses slug expression by recruiting HDAC1 and HDAC2 corepressors to its proximal promoter region [92]. Twist is another important EMT-TF. The overexpression of SIRT3 inhibits EMT and cell metastatic capability by down-regulating twist in ovarian cancer cells [76]. In ovarian cancer, the expression level of HDAC3 was significantly correlated with the expression level of FOXA1. HDAC3 can modulate FOXA1 through the Wnt/β-catenin signaling pathway, and the overexpression of FOXA1 can promote cell proliferation and invasion [93]. HDAC4 can promote the proliferation and migration of epithelial ovarian cancer cells via the repression of p21 on fibrillar collagen matrices [94]. Paired box 8 (PAX8) is a prototype lineage survival oncogene in epithelial ovarian cancer. Furthermore, the PAX8-FGF18 axis promotes cell migration in an autocrine fashion. Class I HDAC inhibition antagonizes PAX8 expression and suppresses ovarian tumor growth and spreading [95].

**Figure 1:**
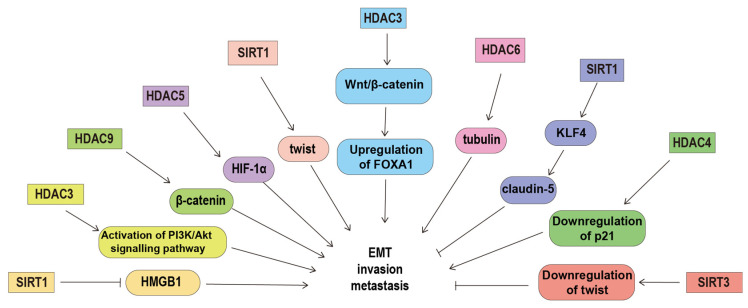
The underlying mechanism of histone deacetylases in regulating ovarian cancer metastasis. HDACs can regulate invasion and metastasis by deacetylating their non-histone substrates (such as tubulin, β-catenin, HMGB1 and KLF4), regulating the expression of p21, claudin-5, FOXA1 and twist, and prompting the activation of the PIAK/Akt signaling pathway. Abbreviations: forkhead box A1, FOXA1; high-motility group box-1 protein, HMGB1; histone deacetylase, HDAC; hypoxia-inducible factor-1α, HIF-1α; Kruppel-like factor 4, KLF4; sirtuins, SIRT; The phosphoinositide 3-kinase, PI3K.

HDACs are also able to regulate cell migration and invasion by regulating the acetylation of their non-histone substrates. The dynamics of microtubules play an important role in ovarian cancer cell migration via affecting the remodeling of the cytoskeleton [96]. HDAC6 can promote ovarian cancer cell motility by deacetylating tubulin [97]. Cortactin, an F-actin-binding protein, also plays important roles in the regulation of cell migration. In ovarian cancer cells, SIRT1 can promote cell migration by deacetylating cortactin [98]. SIRT1 can also activate claudin-5 transcription and maintains the epithelial phenotype of ovarian cancer cells via deacetylating kruppel-like factor 4 [99]. SIRT1 over-expression decreases the expression and acetylation of high-motility group box-1 protein, thus inhibiting ovarian cancer migration, invasion and angiogenesis [100]. β-catenin is the core transcriptional coactivator of the classical Wnt/β-catenin signaling pathway that correlates with the grade, epithelial to mesenchymal transition, chemotherapy resistance, and a poor prognosis in ovarian cancer [101]. The effects of β-catenin on the expression of its target genes depend on its subcellular localization. In non-serous ovarian cancer, HDAC 9 can induce β-catenin translocation to the cytoplasm and decrease the expression of its downstream gene through regulating the acetylation of β-catenin [72]. In addition, up-regulated HDAC5 can promote cell proliferation, migration and invasion by interacting with hypoxia-inducible factor-1α (HIF-1α) and elevating the protein level of HIF-1α [102]. (Figure 1).

As mentioned above, HDACs may participate in the regulation of ovarian cancer metastasis through regulating the activation of EMT and the expression of genes associated with tumor metastasis. Indeed, ovarian cancer cells can enter an intermediate partial EMT state and these cells show cancer stem cell features and are highly aggressive compared with cells in a complete mesenchymal phenotype [103]. Moreover, the intermediate mesenchymal subgroup of ovarian cancer cell lines, rather than the mesenchymal subgroup, has exhibited the strongest migratory and invasive capacities [104]. In summary, ovarian cancer cells may alternately undergo EMT, possibly as a range of states with both epithelial and mesenchymal differentiation, where HDACs may be important regulators.

## 6. Targeting Histone Deacetylases in Ovarian Cancer

### 6.1. Histone Deacetylases Inhibitors

In recent decades, a variety of compounds that are able to block the deacetylase activity of HDACs have been identified. These compounds can inhibit cell invasion and tumor metastasis in vitro and in vivo via promoting the expression of metastasis suppressor genes and inhibiting the expression of pro-metastasis genes [105]. Many synthetic or natural molecules targeting class I, II, and IV HDACs have been developed and characterized. Currently, the class III sirtuin family has also attracted the attention of researchers, and inhibitors targeting these enzymes are being developed. Unlike the class III sirtuin family, the enzymatic activity of class I, II and IV HDACs is dependent on Zn^2+^, so HDACIs which can bind to zinc ions are able to affect the deacetylase activity of HDACs and impair their enzymatic activity [106]. In addition to tumor metastasis, the imbalance between HATs and HDACs may also contribute to tumorigenesis [107]. HDACIs can alter the aberrant acetylation status of proteins found in cancer cells and re-induce the expression of tumor suppressor genes. In addition, cancer cells may be more sensitive to HDACI-induced apoptosis than normal cells, which enhances the therapeutic potential of HDACIs [108].

HDACIs can act only on certain types of HDACs (HDAC isoform-selective inhibitors) or on all types of HDACs (pan-HDAC inhibitors) [109]. Despite their great structural diversity, HDACIs are mainly composed of three main pharmacophore groups, including a zinc-binding group (ZBG), a linker and a CAP-group [110]. Based on the chemical structure of ZBG, HDACIs can be classified into four subclasses: hydroxamic acids, short-chain fatty(aliphatic) acids, benzamides and cyclic peptides [16,111,112]. Hydroxamic acids comprise the largest HDACI category, including TSA, SAHA, panobinostat (LBH589) and belinostat (PDX101) [113]. TSA is the first natural hydroxamate found to inhibit HDACs, and its chemical structure is similar to that of SAHA. However, due to its toxicity, TSA can only be used in laboratory studies [114]. Short-chain fatty acids, such as valproic acid (VPA) and butyric acid, mainly target class I and IIa HDACs [115]. Benzamides and cyclic peptides mainly target class I HDACs [116]. In in vivo experiments, these HDACIs have shown very promising therapeutic effects in the treatment of a variety of tumors. Many in vitro experiments are trying to understand the mechanisms via which these molecules exert their anticancer effects [117]. To date, several HDACIs have been approved by the FDA as anticancer agents, including SAHA, LBH589, PXD101 and FK228 [118]. Additionally, a variety of HDACIs have been investigated in clinical trials, including CS055, quisinostat, MS-275 and 4SC202 [16]. Various alternatives to ZBG, such as hydrazides, catechins and sulfonamides, have shown favorable inhibitory effects on the deacetylase activity of HDAC and deserve further investigation [119,120,121].

### 6.2. Histone Deacetylase Inhibitors in Ovarian Cancer Therapy

As a potent epigenetic suppressor, HDACI reactivates the expression of tumor suppressor genes responsible for apoptosis, cell cycle arrest, and the inhibition of angiogenesis and metastasis [105,106,122]. Multiple transcriptional and non-transcriptional mechanisms are involved in this process [123]. HDACI is a well-tolerated therapeutic agent with good therapeutic efficacy in several hematologic tumors. Numerous preclinical studies have found that HDACI is able to inhibit ovarian cancer cell growth in vitro and in vivo by inhibiting the cell cycle and inducing mitotic defects through histone-mediated and histone-independent interactions [124]. Currently a variety of HDACIs have been investigated in clinical trials involving patients suffering from ovarian cancer (Table 1). HDACIs combined with other chemotherapeutic agents have shown synergistic antitumor effects, which may arise mainly from DNA damage or interference with the DNA damage response (DDR). In general, the antitumor effect of chemotherapy is often limited by the resistance of cancer cells to chemotherapeutic agents. A combination of low-dose HDACIs may reverse chemotherapy resistance through a certain resistance phenotype [125].

HDACs play important roles in the migration and invasion of normal and malignant cells, and inhibition of HDACs can induce the reactivation of tumor metastasis suppressor genes, which in turn inhibits tumor invasion and metastasis. The HDACI apicidin can significantly inhibit the expression of HDAC4 in ovarian cancer SKOV3 cells. Apicidin inhibits the invasion and migration of SKOV3 cells by upregulating the expression of reversion-inducing cysteine-rich protein with Kazal motifs (RECK) and downregulating the expression of MMP2 [137]. CHD4 is a key component of the NuRD complex [138,139]. A high expression of CHD4 in ovarian cancer is associated with tumor metastasis and poor prognosis for patients with ovarian cancer [91]. Studies report that CHD4 plays an important role in cancer by participating in histone deacetylation and PPAR-dependent DNA damage repair. Romidepsin, an HDAC1/2 selective inhibitor, is able to inhibit ovarian cancer metastasis through inhibiting the functions of CHD4 that are mediated by histone deacetylase [91]. Betaglycan is a coreceptor that regulates TGFβ, activin and inhibin signaling. Histone methyltransferase inhibitor 5-aza-2-deoxycytidine and HDACI TSA treatment can synergistically promote the transcription and expression of betaglycan. Although it is not sufficient to restore TGFβ-mediated proliferation inhibition, it is able to significantly inhibit the invasion and migration of ovarian cancer cells [140]. Studies have shown that VPA could significantly inhibit the expression of vascular endothelial growth factor (VEGF) and MMP9, promote the expression of E-cadherin, and inhibit the migration and invasion of ovarian cancer cells in vitro and in vivo [141]. Treatment with HDACI sodium butyrate (NaBu) can restore the expression of E-cadherin in normal cell lines and drug-resistant ovarian cancer cell lines, and partially reverse the EMT program of tumor cells [142]. SAHA can inhibit the growth of paclitaxel-resistant ovarian cancer cells and suppress cell migration by inducing cell cycle arrest, apoptosis and autophagy [143]. There is a crosstalk between DNA methylation and histone deacetylation. HDACIs combined with DNA methyltransferase inhibitors have a synergistic effect on the reactivation of tumor suppressor genes. The combination of TSA and decitabine significantly impairs the migration and invasion capacity of SKOV3 cells through the inhibition of Twist, N-cadherin, MMP-2 and MMP-9, and the induction of E-cadherin [144].

### 6.3. Challenges of HDAC Inhibitors in Cancer Therapy

HDAC is a potential epigenetic target that has attracted considerable attention in the treatment of cancer. HDACIs affect multiple cellular processes such as the cell cycle, cell proliferation, apoptosis and differentiation, and also modulate the immune system [145]. HDACIs have broad effects on chromatin and can modulate the expression of many genes at the same time, not only reactivating some tumor suppressors, but also affecting the expression of many other genes. For example, TSA treatment could promote the expression of survivin by transiently activating the epidermal growth factor receptor/PI3K/AKT cell survival pathway [146]. After treatment with HDACIs, the expression of solute carrier family 6, member 12, is profoundly enhanced, leading to the increased migration and invasion of ovarian cancer cells [147]. Many HDACIs contain hydroxamic acid as the ZBG. However, hydroxamic acid is prone to hydrolysis and glucuronidation, which compromises its potency in vivo [148]. The potential mutagenicity of hydroxamate-based HDACIs is another raised concern limiting their application in cancer therapy [149]. In addition, the adverse effects and cytotoxicity of HDACIs remain serious, which may be related to the lack of subtype selectivity. In general, HDACIs are unable to specifically target certain isoforms in the subclass of HDACs [150]. More selective HDACIs as well as combination therapies may be able to improve their therapeutic efficacy and overcome toxicity and HDACI resistance. HDACIs containing aminobenzamide, another widely used ZBG, have class I selectivity and superior pharmacokinetic properties [151]. Therefore, the search for a new ZBG is beneficial to the development of the next generation of HDACIs with desirable pharmacokinetic profiles and isoform selectivity.

At present, HDACIs are only indicated for hematological cancers. Drug resistance is the major problem limiting the clinical applications of HDACI in solid tumors [152]. HDACIs in combination with other chemotherapeutics have been confirmed to show chemosensitizing or synergistic antitumor efficacy, which may be due to their ability to overcome particular mechanisms associated with drug resistance [111]. However, undesirable drug–drug interactions, pharmacokinetic complexity, drug side-effects and patient compliance limit the effects of drug combination [153]. Designing multitargeted HDAC inhibitors could be the logical way around the drug resistance problem and drug combinations. Over the past few years, many multitargeted HDACIs have been identified. Most of these inhibitors contain hydroxamic acid as a HDAC functional group, which leads to unfavorable pharmacokinetic properties [151]. Therefore, further in-depth studies are needed for the discovery of multitargeted HDACIs with good drug-likeness. Furthermore, the development of a novel ZBG is also one of the potential solutions.

## 7. Discussion

Ovarian cancer is the most lethal gynecological malignant tumor, and metastasis is the major cause of death in patients with ovarian cancer. Studies have shown that in serval human tumor tissues, decreased histone acetylation is significantly correlated with tumor malignancy and tumor invasion. HDACs can reversibly regulate the acetylation of histones and non-histone proteins, and are key modulators of acetylation and epigenetics in cells, providing an attractive target for the treatment of tumor metastasis. Unfortunately, the underlying mechanism of HDACs regulating ovarian cancer metastasis remains unclear. Many researchers have reported how HDACs effect tumor metastasis in various solid tumors including gynecological tumors, which may give us some insights as there are some commonalities among different solid tumors. Currently, HDACIs are often used in combination with other epigenetic inhibitors or commonly used chemotherapeutic agents. Combinations of HDACIs with cytostatic agents (such as paclitaxel and doxorubicin) can significantly improve their antitumor therapeutic efficacy. In addition, the chemosensitization effect of HDACIs is also related to the combination of drugs. Besides HDACs, other epigenetic enzymes are abnormally expressed in ovarian cancer and participate in ovarian cancer metastasis [154,155,156], which indicates that the combination of HDACIs and inhibitors of these epigenetic enzymes may improve outcomes for patients. In ovarian cancer, many preclinical studies have found that both single-agent HDACIs and the combination of HDACIs with standard chemotherapeutic drugs have significant inhibitory effects on the cell proliferation and metastasis of ovarian cancer [136,157]. However, recent studies have demonstrated that that HDACIs can induce the expression of interleukin 8 in ovarian cancer cells, which may promote the progression of ovarian cancer by promoting cell survival, proliferation, angiogenesis and metastasis [158]. Therefore, more in vitro and in vivo studies are needed to better understand the mechanism of HDACIs in ovarian cancer therapy and the optimal combination of HDACIs with conventional chemotherapy.

Over the past decades, various HDACIs have been identified and their promising antitumor therapeutic efficacy has been confirmed via studies in vivo and in vitro. Five of these HDACIs (SAHA, FK-288, PXD-101, LBH589 and CS055) are approved as antitumor agents by the FDA, and multiple HDACIs are undergoing clinical trials. Although HDACIs have promising clinical efficacy in some hematological tumors, multiple HDACIs fail to show activity in serval cancer types, especially in solid tumors [159,160]. As mentioned above, drug resistance and toxicity are two major issues that limit the clinical applications of HDACIs. Multiple mechanisms are involved in the resistance of HDACIs, including but not limited to the activation of signaling pathways such as CDK and AKT [41]. Since not all HDACs isoforms are overexpressed in ovarian cancer, single-isoform selective inhibitors can help to overcome toxicity and avoid possible side effects. Currently, most HDACIs bind HDAC in the same packet and these HDACIs are unable to specifically targeting certain isoforms in the subclass of HDACs [150]. The structural determination of all HDAC isoforms along with the development of computational techniques such as molecular modeling, cheminformatics, and machine learning can be helpful in the design of isoform-selective HDACIs. More selective HDACIs as well as combination therapies may be able to improve the therapeutic efficacy and overcome toxicity and HDACI resistance.

## Figures and Tables

**Table 1:** Clinical trials of histone deacetylase inhibitors treating ovarian cancer.

HDAC Inhibitor	Drugs in Combination	Cancer Type	Clinical Trial
Belinostat	Carboplatin and/or paclitaxel	Solid tumors including ovarian cancer	Phase I [126]
Belinostat		Metastatic or recurrent platinum-resistant epithelial ovarian cancer	Phase II [127]
Belinostat,	Carboplatin and paclitaxel	Previously treated ovarian cancer	Phase II [128]
Belinostat	Carboplatin	Platinum-resistant ovarian cancer	Phase II [129]
VPA	Oral etoposide	Platinum-resistant ovarian cancer	Phase II [130]
VPA	Carboplatin and Azacitidine	Cancers including ovarian cancer	NCT00529022 [131]
Vorinostat		Persistent or recurrent epithelial ovarian	Phase II [132]
Panobinostat	Gemcitabine	Solid tumors including ovarian cancer	Phase I [133]
Vorinostat	Paclitaxel and carboplatin	Advanced-stage ovarian carcinoma	Phase II [134]
Valproate	Hydralazine	Cancers including ovarian cancer	Phase II [135]
Vorinostat	Carboplati and gemcitabine	Recurrent, platinum-sensitive epithelial ovarian cancer	Phase I [136]
Belinostat	Talazoparib	Metastatic ovarian cancer	NCT04703920
Belinostat	Ribociclib	Recurrent ovarian cancer	NCT04315233
Entinostat	Avelumab and placebo	Advanced epithelial ovarian cancer	NCT02915523

## Data Availability

Not applicable.
